# Karl Landsteiner (1868–1943): A Versatile Blood Scientist

**DOI:** 10.7759/cureus.68903

**Published:** 2024-09-07

**Authors:** Noor Haslina Mohd Noor, Mat Jusoh Siti Asmaa

**Affiliations:** 1 Department of Hematology, Universiti Sains Malaysia, School of Medical Sciences, Kota Bharu, MYS

**Keywords:** blood group system, blood typing, immunohematology, polio, transfusion medicine

## Abstract

Karl Landsteiner (1868-1943) was an Austrian-American biologist, physician, and immunologist whose groundbreaking discoveries revolutionized the fields of transfusion medicine, immunology, and virology. His most famous work was the identification of the ABO blood group system in 1901, which explained the causes of transfusion reactions and laid the foundation for safe blood transfusions. This discovery earned him the Nobel Prize in Physiology or Medicine in 1930. Landsteiner continued his research, identifying the MN and P blood group system in 1927 and the Rh blood group system in 1940, which addressed the complexities of the Hemolytic Disease of the Fetus and Newborn (HDFN). His work on the poliovirus in 1908, in collaboration with Erwin Popper, established the infectious nature of the disease and laid the groundwork for future vaccine development. Landsteiner's versatility as a scientist is evident in the breadth of his work, spanning hematology, immunology, and virology. His ability to navigate diverse fields and see connections between them allowed him to make pioneering discoveries that have had a lasting impact on medical practice, particularly in blood transfusion, organ transplantation, and immunotherapy. Landsteiner's legacy as the "Father of Transfusion Medicine" is reinforced by the standardization of blood typing procedures, which have saved millions of lives worldwide.

## Introduction and background

Early life and education

Karl Landsteiner's early life and education significantly shaped his scientific career. Karl Landsteiner was born on June 14, 1868, in Vienna, Austria, and was an Austrian-American biologist, physician, and immunologist. His father, Leopold Landsteiner, was a prominent journalist and editor, but he passed away when Karl was only six years old. Since Karl lost his father at a young age, this fostered a close bond with his mother, Fanny Hess Landsteiner. This nurturing environment likely encouraged his intellectual pursuits [1].

Landsteiner attended the University of Vienna, where he studied medicine, earning his medical degree in 1891. During his university years, he was particularly influenced by the work of his professors in chemistry and pathology, which steered his interest toward biomedical research. He published an article on diet's influence on blood composition, showcasing his early interest in blood chemistry. Following his graduation, he sought to deepen his knowledge of chemistry by working in renowned laboratories across Europe, including those of Emil Fischer and Eugen Bamberger, which provided him with a strong foundation in both chemistry and immunology [2]. Emil Fischer is a German Nobel laureate in Chemistry, who found the essential biological molecules, sugar, and purine structure, and introduced the "lock and key" model to describe enzyme specificity [3]. Eugen Bamberger, another renowned chemist, made significant contributions to organic chemistry, particularly in the study of aromatic compounds and oxidation reactions, which led to advancements in chemical synthesis methodologies [4]. The training Landsteiner received equipped him with a unique skill set that bridged the gap between chemistry and biology.

**Figure 1:**
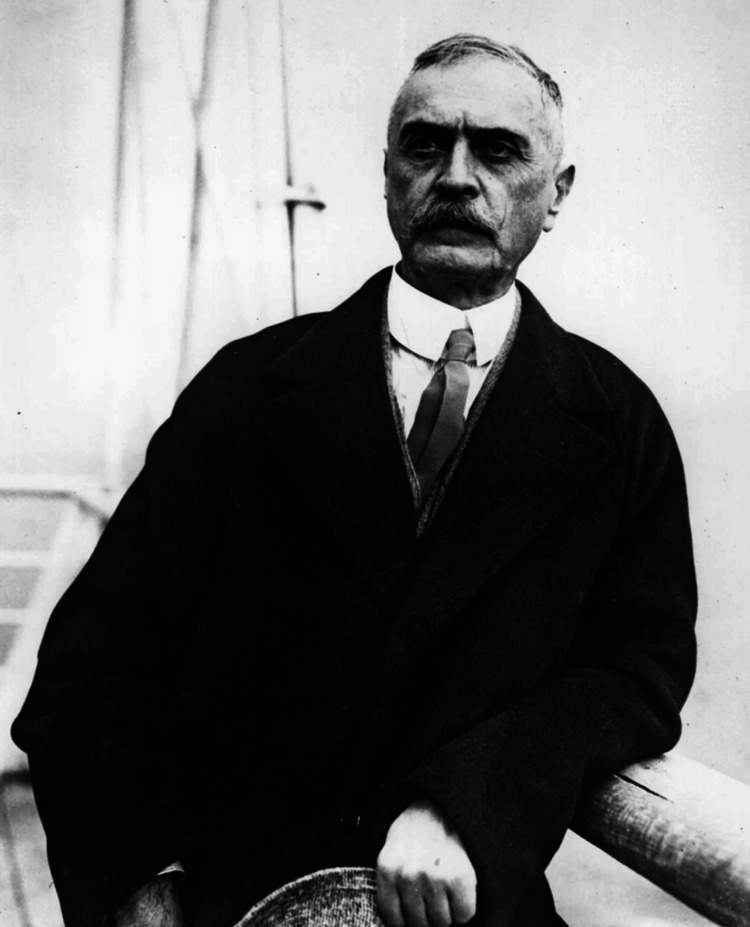
Portrait of Karl Landsteiner (1868–1943), the “Father of Transfusion Medicine” Source: Encyclopedia Britannica, 22 Jun 2024 [5]

Upon returning to Vienna, Landsteiner worked under prominent figures like Max von Gruber at the Hygiene Institute, where he focused on immunity and antibodies. His position as an assistant in the University Department of Pathological Anatomy further honed his skills in morbid physiology, allowing him to explore the mechanisms of immune responses. This combination of medical training, research experience, and mentorship laid the groundwork for his later discoveries, including the ABO blood group system and other significant immunological findings. He is considered the Father of Transfusion Medicine, and his work has saved millions of lives (Figure 1) [5].

## Review

Discovery of the ABO blood group system (1901)

The discovery of the ABO blood group system marked a pivotal moment in medical science, revolutionizing the field of transfusion medicine and laying the foundation for modern hematology. This breakthrough was the result of meticulous research conducted by Karl Landsteiner in the early twentieth century. Landsteiner's most famous and impactful work began in 1900 when he discovered the major human blood groups A, B, O, and AB [5]. Before the discovery of blood groups, blood transfusions were fraught with danger. Physicians knew that transfusions sometimes led to fatal reactions, but the reasons were unclear. The prevailing belief was that all human blood was essentially the same, and when transfusions failed, it was often attributed to technical errors or patient frailty rather than fundamental biological incompatibility. Landsteiner's work involved a series of experiments in which he mixed blood samples from different people and observed the reactions. He hypothesized that the reactions were due to differences in the blood of different individuals. Landsteiner's key observation was that when blood from different individuals was mixed, it sometimes clumped together, a phenomenon known as agglutination [2]. He noticed that this agglutination occurred in a predictable pattern depending on the specific blood samples used. Based on his experiments, Landsteiner proposed that human blood could be divided into distinct groups. He discovered in 1901 that an interaction between blood and blood serum, which contains the antibodies, is what causes the agglutination. From these observations, he identified three distinct blood groups: A, B, and O. A blood group has the A antigen on RBCs and anti-B antibodies in the plasma. B blood group has B antigen on RBCs and anti-A antibodies in the plasma. While O blood group lacks A and B antigens on RBCs and has both anti-A and anti-B antibodies in the plasma. Identifying these groups explained why some transfusions succeeded while others failed: incompatible blood types could trigger agglutinations [6].

In 1902, Alfred von Decastello, one of Landsteiner's colleagues, and his student Adriano Sturli discovered the fourth blood group, AB, which includes both A and B antigens on RBCs but neither anti-A or anti-B antibodies in the plasma. The finding completed the ABO blood group system as we know it today. This cutting-edge discovery has changed the process of blood transfusion, making it much less risky by ensuring compatibility between donor and recipient. The first successful blood transfusion was carried out by Reuben Ottenberg, an American physician using the ABO blood group system at Mount Sinai Hospital in New York in 1907. Following this, the first blood typing test was established in 1910, which led to an official blood banking service in the 1930s [7]. Landsteiner's following findings on the ‘AB’ as universal recipient and ‘O’ as universal donors have greatly impacted the area of transfusion medicine. In 1930, Karl Landsteiner was awarded the ‘Nobel Prize in Physiology or Medicine’ and was called the ‘Father of Transfusion Medicine’ due to his pioneered works [8].

Identification of the MN blood group system (1927)

After his work on the ABO system, Landsteiner continued to explore other blood group antigens while working at the Rockefeller Institute for Medical Research in New York City, now known as Rockefeller University. In 1927, through his work with Philip Levine, an American immunohematologist, he discovered the MN blood group system. This blood group system is defined by the presence of two antigens, M and N, on the surface of RBCs. The MN system is one of the many human blood group systems that have been identified, and it is determined by a pair of co-dominant alleles, LM and LN, located on chromosome 4 [9]. They did some experiments to systematically test blood samples to identify potential new antigens beyond the already known ABO system. To do this, Landsteiner and Levine collected blood samples from various individuals, including themselves. Then, they separated the serum (the liquid part of the blood) and RBCs from each sample. They then tested these samples for agglutination (clumping of RBCs) reactions, which occur when antibodies in the serum bind to specific antigens on the surface of RBCs. Through their systematic testing, Levine and Landsteiner identified two distinct antigens on RBCs, which they named M and N. These antigens are inherited in a Mendelian fashion, with individuals being classified as M, N, or MN [5]. They discovered that individuals could possess either the M antigen, the N antigen, or both (MN), leading to the classification of blood into three types: M, N, and MN. Although the MN system is less significant clinically compared to ABO and Rh, it has been crucial in studies of human genetics, anthropology, and paternity testing.

Identification of the P blood group system (1927)

In the same year, 1927, Landsteiner, along with Levine and Alexander Wiener, discovered the P blood group system. The P system involves antigens P, P1, and Pk. This discovery was important for understanding transfusion reactions and certain hemolytic conditions such as Paroxysmal Cold Hemoglobinuria (PCH). PCH is a rare type of autoimmune hemolytic anemia often associated with viral infections, particularly in children. In PCH, the body produces an antibody known as the Donath-Landsteiner antibody, which is a biphasic hemolysin. This antibody attaches to the P antigen on RBCs at cold temperatures (like in the extremities during cold exposure) and then causes red blood cell lysis when the blood is warmed to body temperature [10]. The presence of the P antigen on RBCs is critical for this antibody's activity. Although not as common as reactions involving the ABO or Rh systems, transfusion reactions can occur due to incompatibility in the P blood group system. Individuals who lack the Pk antigen (extremely rare) may produce anti-Pk antibodies. If such a person receives blood that contains the Pk antigen, a hemolytic transfusion reaction could occur. This is why careful blood matching is essential in transfusion medicine, even for less common antigens like those in the P system. In a very rare instance, a woman with a ‘null’ P phenotype (who lacks all P system antigens) is at risk of developing anti-P antibodies. If these women become pregnant with a fetus expressing P antigens, the maternal antibodies can cross the placenta and attack fetal RBCs, leading to hemolysis and potentially resulting in spontaneous abortion or severe fetal anemia [11]. The P blood group system, like MN, is not as critical due to low prevalence in transfusion practice compared to ABO or Rh, but it added to the layers of understanding of the complexity of human blood antigens.

Identification of the Rhesus (Rh) blood group system (1940)

The discovery and identification of the Rh blood group system is a significant chapter in the history of immunohematology. Before the discovery of the Rh factor, physicians were puzzled when a noticeable percentage of patients still experienced hemolytic reactions after blood transfusion. This condition was prominent in a pregnant woman when their fetus had a condition known as hemolytic disease of the fetus and newborn (HDFN), also called erythroblastosis fetalis. This disease caused severe anemia, jaundice, and often death in the fetus and newborns. While it was known that HDFN was related to blood group incompatibilities, particularly between the mother and fetus, the exact cause was not understood. The human Rh blood group system was discovered in 1940 by Landsteiner, and his colleague Alexander S. Wiener. This discovery arose from experiments that involved immunizing rabbits with the RBCs of rhesus monkeys (Macaca mulatta), which led to the production of antibodies in the rabbits. The Macaca species was preferred due to 93-98% of genetic resemblance to humans, which provided a reliable model for studying human immunology [12]. In the experiment, Landsteiner and Wiener injected the RBCs of rhesus monkeys into rabbits. The rabbits’ immune systems produced antibodies against these foreign cells. To their surprise, when they tested these antibodies against human RBCs, they observed that the serum caused agglutination in approximately 85% of human blood samples. This indicated the presence of a previously unknown antigen on the surface of these human RBCs. They named this new antigen the Rh factor, after the rhesus monkey from which it was first identified. The presence or absence of this antigen classified people as Rh-positive or Rh-negative [13].

The discovery of the Rh factor provided a critical explanation for the hemolytic disease of the newborn. The disease occurs when a Rh-negative mother carries an Rh-positive fetus. During pregnancy or childbirth, fetal RBCs can enter the mother’s bloodstream, causing her immune system to produce anti-Rh antibodies. In subsequent pregnancies, if the fetus is again Rh-positive, these maternal antibodies can cross the placenta and attack the fetal RBCs, leading to hemolysis and the associated symptoms of HDFN.

After the initial discovery by Landsteiner and Wiener, further research elucidated the complexity of the Rh system, which includes multiple antigens such as D, C, c, E, and e. The D antigen is the most immunogenic and clinically significant, which is why the Rh factor is often specifically referred to as the presence or absence of the D antigen. The discovery of the Rh blood group system confers great implications that became a legacy in transfusion medicine. The identification of the Rh factor led to the development of Rho(D) immune globulin (RhoGAM) in the 1960s, a treatment that can prevent Rh-negative mothers from developing antibodies against Rh-positive fetal cells. This treatment has drastically reduced the incidence of HDFN. The Rh system also became a crucial factor in blood transfusion compatibility, with Rh-positive and Rh-negative designations becoming standard in blood typing to prevent transfusion reactions. Karl Landsteiner’s and Alexander Wiener’s work, along with subsequent research, solidified the Rh system's role as one of the most important blood group systems in medicine. Landsteiner’s pioneered works on the Rh factors earned him the 1946 Albert Lasker Clinical Medical Research Award [7].

Research on poliomyelitis (1908)

Landsteiner also made significant contributions to the understanding of poliomyelitis in the early twentieth century. In collaboration with Erwin Popper, he is credited with discovering the poliovirus in 1908. Landsteiner and Popper's research began with the autopsy of a nine-year-old boy who died from polio. They injected a suspension of his spinal cord into monkeys, which subsequently developed symptoms consistent with poliomyelitis. Their experiments confirmed that the poliovirus belonged to a group of filterable microorganisms, a crucial step in identifying the etiology of the disease. This work established the infectious nature of the disease and laid the groundwork for future vaccine development [14]. Landsteiner's findings were pivotal in the eventual development of polio vaccines. Although he noted in 1912 that creating a vaccine would be challenging, his work set the stage for future researchers, leading to the successful development of vaccines by Jonas Salk and Albert Sabin in the mid-20th century [15].

Versatility and legacy

Karl Landsteiner's versatility as a scientist is evident in the breadth of his work, spanning hematology, immunology, and virology. His ability to see connections between different scientific disciplines allowed him to make pioneering discoveries that have had a lasting impact on medicine. Landsteiner's dedication to research was recognized by the awarding of the Nobel Prize in Physiology or Medicine in 1930 for his discovery of the ABO blood groups [1]. Landsteiner began his career in pathology but quickly expanded his focus to include immunology, hematology, and virology. His ability to navigate these diverse fields was driven by a keen interest in how the body's defense mechanisms work and how they can be harnessed to improve medical care. For instance, his work on the ABO blood group system emerged from his foundational studies in serology, the study of blood serum and its reactions, which was itself an intersection of pathology and immunology. His discovery of the Rh factor, in collaboration with Alexander S. Wiener, was another testament to his versatility. At a time when the understanding of blood transfusions was still developing, Landsteiner's work provided critical insights into why some transfusions failed even when blood types were matched, thereby extending his influence within hematology.

Beyond hematology, Landsteiner made groundbreaking contributions to virology. His work with Erwin Popper on the poliovirus opened new avenues in the study of viral diseases, laying the groundwork for future vaccine development. This discovery was particularly significant in the early 20th century when polio was a major public health threat. Landsteiner's ability to identify the poliovirus underscored his capacity to address imperative medical challenges of his time, often leading to solutions that would benefit future generations. Landsteiner's research on hapten, a small molecule that can elicit an immune response only when attached to a larger protein, demonstrated his deep understanding of immunological principles [16]. This work was crucial in advancing the field of immunology, particularly in understanding how the immune system recognizes and reacts to foreign substances. It is a concept still central to modern immunology, with applications ranging from vaccine development to the design of immunotherapies for cancer.

Karl Landsteiner's discoveries have had a lasting impact on medical practice, particularly in the realms of blood transfusion, organ transplantation, and immunotherapy. The ABO blood group system remains a fundamental aspect of transfusion medicine, and the identification of the Rh factor continues to be crucial in prenatal care. His contributions have enabled the standardization of blood typing procedures, saving millions of lives in surgeries, childbirth, and trauma care. Moreover, Landsteiner’s work laid the foundation for organ transplantation [2]. Understanding blood types and immune responses is critical in preventing organ rejection, a concept that directly stems from his research on blood groups and antigens. His legacy in this area is reflected in the success of organ transplants, which have become routine life-saving procedures.

Landsteiner’s intellectual curiosity and broad approach to science have inspired countless researchers to adopt a multidisciplinary perspective. His career serves as a powerful example of how crossing traditional boundaries between scientific disciplines can lead to groundbreaking discoveries. In many ways, Landsteiner anticipated the modern trend toward interdisciplinary research, where breakthroughs often occur at the intersection of different fields. His legacy is also educational, showing that significant scientific advancements often require a willingness to explore unfamiliar territories, challenge existing paradigms, and integrate knowledge from various domains. As such, Landsteiner remains an iconic figure in science, not only for his discoveries but also for his approach to research.

## Conclusions

Karl Landsteiner's contributions to medical science, particularly in transfusion medicine and immunology, are profound and impactful. His identification of the ABO blood group system in 1901 marked a pivotal advancement that transformed blood transfusions from a risky procedure into a safe and standard practice, significantly reducing the incidence of transfusion reactions. This foundational work was complemented by his discoveries of the Rh, MN, and P blood group systems, which further enhanced the safety and understanding of blood compatibility, particularly in prenatal care and organ transplantation. Landsteiner's work on the poliovirus laid the foundation for the current vaccine research. His interdisciplinary approach led to groundbreaking discoveries and inspired modern scientific collaboration.

Overall, Landsteiner's legacy as the "Father of Transfusion Medicine" is solidified by the lasting impact of his work on blood typing standardization and the development of safe medical practices that have saved millions of lives globally. Landsteiner's work continues to inspire modern science, especially as interdisciplinary research becomes crucial for addressing complex medical challenges. His legacy is a reminder that the pursuit of knowledge, driven by curiosity and supported by collaboration across disciplines, can lead to innovations that fundamentally change the course of medical science and improve human health on a global scale.
